# 2-(*p*-Tol­yloxy)pyrimidine

**DOI:** 10.1107/S1600536809026610

**Published:** 2009-07-15

**Authors:** Nasir Shah Bakhtiar, Zanariah Abdullah, Seik Weng Ng

**Affiliations:** a Department of Chemistry, University of Malaya, 50603 Kuala Lumpur, Malaysia

## Abstract

In the title compound, C_11_H_10_N_2_O, the aromatic rings make a dihedral angle of 76.3 (1)°. The C—O—C angle at the ether atom is widened to 117.79 (9)°.

## Related literature

For 2-phenoxy­pyrimidine, see: Shah Bakhtiar *et al.* (2009 ▶).
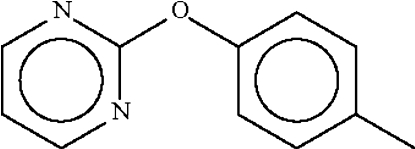


## Experimental

### 

#### Crystal data

C_11_H_10_N_2_O
*M* *_r_* = 186.21
Orthorhombic, 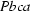

*a* = 11.2918 (2) Å
*b* = 7.2275 (1) Å
*c* = 23.3359 (5) Å
*V* = 1904.48 (6) Å^3^
*Z* = 8
Mo *K*α radiation
μ = 0.09 mm^−1^
*T* = 153 K
0.35 × 0.35 × 0.35 mm

#### Data collection

Bruker SMART APEX diffractometer
Absorption correction: none
12308 measured reflections
2189 independent reflections
1789 reflections with *I* > 2σ(*I*)
*R*_int_ = 0.025

#### Refinement

*R*[*F* ^2^ > 2σ(*F* ^2^)] = 0.038
*wR*(*F* ^2^) = 0.109
*S* = 1.02
2189 reflections
128 parameters
H-atom parameters constrained
Δρ_max_ = 0.19 e Å^−3^
Δρ_min_ = −0.20 e Å^−3^

### 

Data collection: *APEX2* (Bruker, 2008 ▶); cell refinement: *SAINT* (Bruker, 2008 ▶); data reduction: *SAINT*; program(s) used to solve structure: *SHELXS97* (Sheldrick, 2008 ▶); program(s) used to refine structure: *SHELXL97* (Sheldrick, 2008 ▶); molecular graphics: *X-SEED* (Barbour, 2001 ▶); software used to prepare material for publication: *publCIF* (Westrip, 2009 ▶).

## Supplementary Material

Crystal structure: contains datablocks global, I. DOI: 10.1107/S1600536809026610/bt2996sup1.cif

Structure factors: contains datablocks I. DOI: 10.1107/S1600536809026610/bt2996Isup2.hkl

Additional supplementary materials:  crystallographic information; 3D view; checkCIF report

## Figures and Tables

**Fig. 1.**
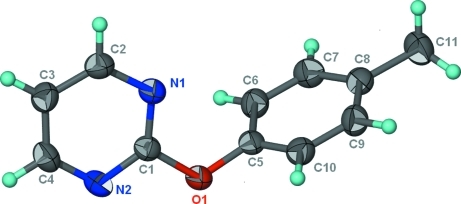
Anisotropic displacement ellipsoid plot (Barbour, 2001) of C11H10N2O at the 70% probability level; hydrogen atoms are drawn as spheres of arbitrary radius.

## supplementary crystallographic information

### Experimental

*p*-Cresol (2.16 g, 20 mmol) and sodium hydroxide (0.80 g, 20 mmol) were dissolved in water (50 ml) and to the solution was added 2-chloropyridimidine (2.30 g, 20 mmol) dissolved in THF (50 ml). The mixture was heated for 4 h. Water was added and the organic phase was extracted with chloroform. The chloroform solution was dried over sodium sulfate; slow evaporation led to the formation of colorless crystals.

### Refinement

H-atoms were placed in calculated positions (C—H 0.95–0.98 Å) and were included in the refinement in the riding model approximation, with *U*(H) set to 1.2–1.5*U*(C).

### Crystal data

**Table:**

C_11_H_10_N_2_O	*F*(000) = 784
*M**_r_* = 186.21	*D*_x_ = 1.299 Mg m^−^^3^
Orthorhombic, *P**b**c**a*	Mo *K*α radiation, λ = 0.71073 Å
Hall symbol: -P 2ac 2ab	Cell parameters from 3922 reflections
*a* = 11.2918 (2) Å	θ = 2.8–28.2°
*b* = 7.2275 (1) Å	µ = 0.09 mm^−^^1^
*c* = 23.3359 (5) Å	*T* = 153 K
*V* = 1904.48 (6) Å^3^	Irregular, colorless
*Z* = 8	0.35 × 0.35 × 0.35 mm

### Data collection

**Table:**

Bruker SMART APEX diffractometer	1789 reflections with *I* > 2σ(*I*)
Radiation source: fine-focus sealed tube	*R*_int_ = 0.025
graphite	θ_max_ = 27.5°, θ_min_ = 1.8°
ω scans	*h* = −14→13
12308 measured reflections	*k* = −9→9
2189 independent reflections	*l* = −30→30

### Refinement

**Table:**

Refinement on *F*^2^	Primary atom site location: structure-invariant direct methods
Least-squares matrix: full	Secondary atom site location: difference Fourier map
*R*[*F*^2^ > 2σ(*F*^2^)] = 0.038	Hydrogen site location: inferred from neighbouring sites
*wR*(*F*^2^) = 0.109	H-atom parameters constrained
*S* = 1.02	*w* = 1/[σ^2^(*F*_o_^2^) + (0.0517*P*)^2^ + 0.6197*P*] where *P* = (*F*_o_^2^ + 2*F*_c_^2^)/3
2189 reflections	(Δ/σ)_max_ = 0.001
128 parameters	Δρ_max_ = 0.19 e Å^−^^3^
0 restraints	Δρ_min_ = −0.20 e Å^−^^3^

### Fractional atomic coordinates and isotropic or equivalent isotropic displacement parameters (Å^2^)

**Table:**

	*x*	*y*	*z*	*U*_iso_*/*U*_eq_	
O1	0.27705 (7)	0.18284 (13)	0.40394 (4)	0.0310 (2)	
N1	0.38531 (9)	0.28051 (15)	0.48203 (4)	0.0294 (2)	
N2	0.17358 (8)	0.27108 (15)	0.48092 (4)	0.0303 (3)	
C1	0.28062 (9)	0.24949 (16)	0.45862 (5)	0.0253 (3)	
C2	0.38117 (11)	0.34394 (19)	0.53603 (5)	0.0330 (3)	
H2	0.4534	0.3703	0.5552	0.040*	
C3	0.27601 (11)	0.37220 (19)	0.56462 (5)	0.0334 (3)	
H3	0.2743	0.4166	0.6029	0.040*	
C4	0.17305 (11)	0.33291 (18)	0.53495 (5)	0.0332 (3)	
H4	0.0992	0.3505	0.5536	0.040*	
C5	0.38237 (10)	0.18523 (17)	0.37209 (5)	0.0272 (3)	
C6	0.42753 (11)	0.35123 (18)	0.35269 (5)	0.0319 (3)	
H6	0.3916	0.4650	0.3633	0.038*	
C7	0.52621 (11)	0.34878 (18)	0.31740 (5)	0.0335 (3)	
H7	0.5580	0.4625	0.3040	0.040*	
C8	0.57990 (11)	0.18345 (18)	0.30110 (5)	0.0305 (3)	
C9	0.53213 (11)	0.01971 (18)	0.32187 (5)	0.0339 (3)	
H9	0.5677	−0.0946	0.3115	0.041*	
C10	0.43334 (11)	0.01911 (18)	0.35755 (5)	0.0318 (3)	
H10	0.4018	−0.0940	0.3716	0.038*	
C11	0.68626 (13)	0.1829 (2)	0.26196 (6)	0.0416 (3)	
H11A	0.7585	0.1985	0.2847	0.062*	
H11B	0.6796	0.2848	0.2344	0.062*	
H11C	0.6898	0.0650	0.2413	0.062*	

### Atomic displacement parameters (Å^2^)

**Table:**

	*U*^11^	*U*^22^	*U*^33^	*U*^12^	*U*^13^	*U*^23^
O1	0.0222 (4)	0.0426 (5)	0.0281 (4)	−0.0027 (4)	−0.0013 (3)	−0.0065 (4)
N1	0.0229 (5)	0.0361 (6)	0.0291 (5)	−0.0004 (4)	−0.0016 (4)	−0.0035 (4)
N2	0.0215 (5)	0.0360 (6)	0.0335 (5)	−0.0015 (4)	0.0020 (4)	0.0026 (4)
C1	0.0239 (6)	0.0248 (6)	0.0271 (6)	0.0001 (4)	−0.0007 (4)	0.0011 (4)
C2	0.0296 (6)	0.0397 (7)	0.0298 (6)	−0.0048 (5)	−0.0025 (5)	−0.0034 (5)
C3	0.0369 (7)	0.0353 (7)	0.0279 (6)	−0.0040 (5)	0.0048 (5)	−0.0026 (5)
C4	0.0281 (6)	0.0374 (7)	0.0343 (6)	−0.0006 (5)	0.0085 (5)	0.0017 (5)
C5	0.0219 (5)	0.0369 (7)	0.0227 (5)	−0.0008 (5)	−0.0028 (4)	−0.0028 (5)
C6	0.0326 (6)	0.0305 (6)	0.0327 (6)	0.0034 (5)	−0.0003 (5)	−0.0022 (5)
C7	0.0354 (7)	0.0317 (6)	0.0333 (6)	−0.0033 (5)	0.0002 (5)	0.0032 (5)
C8	0.0287 (6)	0.0371 (7)	0.0256 (6)	−0.0010 (5)	−0.0008 (5)	−0.0018 (5)
C9	0.0356 (7)	0.0309 (6)	0.0352 (7)	0.0024 (5)	0.0045 (5)	−0.0038 (5)
C10	0.0336 (6)	0.0305 (6)	0.0313 (6)	−0.0039 (5)	0.0017 (5)	−0.0016 (5)
C11	0.0368 (7)	0.0461 (8)	0.0420 (8)	−0.0015 (6)	0.0096 (6)	−0.0012 (6)

### Geometric parameters (Å, °)

**Table:**

O1—C1	1.3644 (14)	C6—C7	1.3856 (18)
O1—C5	1.4026 (14)	C6—H6	0.9500
N1—C1	1.3215 (14)	C7—C8	1.3928 (18)
N1—C2	1.3419 (16)	C7—H7	0.9500
N2—C1	1.3252 (15)	C8—C9	1.3880 (18)
N2—C4	1.3376 (17)	C8—C11	1.5090 (17)
C2—C3	1.3773 (18)	C9—C10	1.3919 (17)
C2—H2	0.9500	C9—H9	0.9500
C3—C4	1.3826 (18)	C10—H10	0.9500
C3—H3	0.9500	C11—H11A	0.9800
C4—H4	0.9500	C11—H11B	0.9800
C5—C10	1.3740 (17)	C11—H11C	0.9800
C5—C6	1.3801 (17)		
			
C1—O1—C5	117.79 (9)	C7—C6—H6	120.6
C1—N1—C2	114.53 (10)	C6—C7—C8	121.55 (12)
C1—N2—C4	114.44 (10)	C6—C7—H7	119.2
N1—C1—N2	129.31 (11)	C8—C7—H7	119.2
N1—C1—O1	118.23 (10)	C9—C8—C7	117.84 (11)
N2—C1—O1	112.45 (10)	C9—C8—C11	121.23 (12)
N1—C2—C3	122.38 (11)	C7—C8—C11	120.94 (12)
N1—C2—H2	118.8	C8—C9—C10	121.53 (12)
C3—C2—H2	118.8	C8—C9—H9	119.2
C2—C3—C4	116.87 (12)	C10—C9—H9	119.2
C2—C3—H3	121.6	C5—C10—C9	118.73 (12)
C4—C3—H3	121.6	C5—C10—H10	120.6
N2—C4—C3	122.47 (11)	C9—C10—H10	120.6
N2—C4—H4	118.8	C8—C11—H11A	109.5
C3—C4—H4	118.8	C8—C11—H11B	109.5
C10—C5—C6	121.59 (11)	H11A—C11—H11B	109.5
C10—C5—O1	118.38 (11)	C8—C11—H11C	109.5
C6—C5—O1	119.86 (11)	H11A—C11—H11C	109.5
C5—C6—C7	118.75 (12)	H11B—C11—H11C	109.5
C5—C6—H6	120.6		
			
C2—N1—C1—N2	−0.4 (2)	C1—O1—C5—C6	−71.51 (14)
C2—N1—C1—O1	−179.29 (11)	C10—C5—C6—C7	0.44 (18)
C4—N2—C1—N1	−0.13 (19)	O1—C5—C6—C7	−174.79 (10)
C4—N2—C1—O1	178.78 (10)	C5—C6—C7—C8	0.27 (19)
C5—O1—C1—N1	−12.16 (16)	C6—C7—C8—C9	−0.69 (19)
C5—O1—C1—N2	168.79 (10)	C6—C7—C8—C11	179.10 (12)
C1—N1—C2—C3	0.64 (19)	C7—C8—C9—C10	0.42 (19)
N1—C2—C3—C4	−0.3 (2)	C11—C8—C9—C10	−179.37 (12)
C1—N2—C4—C3	0.51 (18)	C6—C5—C10—C9	−0.70 (18)
C2—C3—C4—N2	−0.3 (2)	O1—C5—C10—C9	174.60 (10)
C1—O1—C5—C10	113.10 (12)	C8—C9—C10—C5	0.26 (19)

## References

[bb1] Barbour, L. J. (2001). *J. Supramol. Chem.* **1**, 189–191.

[bb2] Bruker (2008). *APEX2* and *SAINT* Bruker AXS Inc., Madison, Wisconsin, USA.

[bb3] Shah Bakhtiar, N., Abdullah, Z. & Ng, S. W. (2009). *Acta Cryst.* E**65**, o114. 10.1107/S1600536808041196 PMC2968036 21581576

[bb4] Sheldrick, G. M. (2008). *Acta Cryst.* A**64**, 112–122. 10.1107/S0108767307043930 18156677

[bb5] Westrip, S. P. (2009). *publCIF* In preparation.

